# Supplementary material for: Intraspecific sequence variation and complete genomes refine the identification of rapidly evolved regions in humans
Source: bioRxiv. 2025 Oct 21:2025.10.20.683446. Preprint. [Version 1] doi: 10.1101/2025.10.20.683446 (PMC12633414; doi:10.1101/2025.10.20.683446)
Supplement: 1 [file NIHPP2025.10.20.683446V1-supplement-1.pdf]

bioRxiv preprint doi: https://doi.org/10.1101/2025.10.20.683446; this version posted October 21, 2025. The copyright holder for this preprint (which was not certified by peer review) is the author/funder, who has granted bioRxiv a license to display the preprint in perpetuity. It is made available under aCC-BY 4.0 International license.

Supplementary Figures

bioRxiv preprint doi: https://doi.org/10.1101/2025.10.20.683446; this version posted October 21, 2025. The copyright holder for this preprint (which was not certified by peer review) is the author/funder, who has granted bioRxiv a license to display the preprint in perpetuity. It is made available under aCC-BY 4.0 International license.

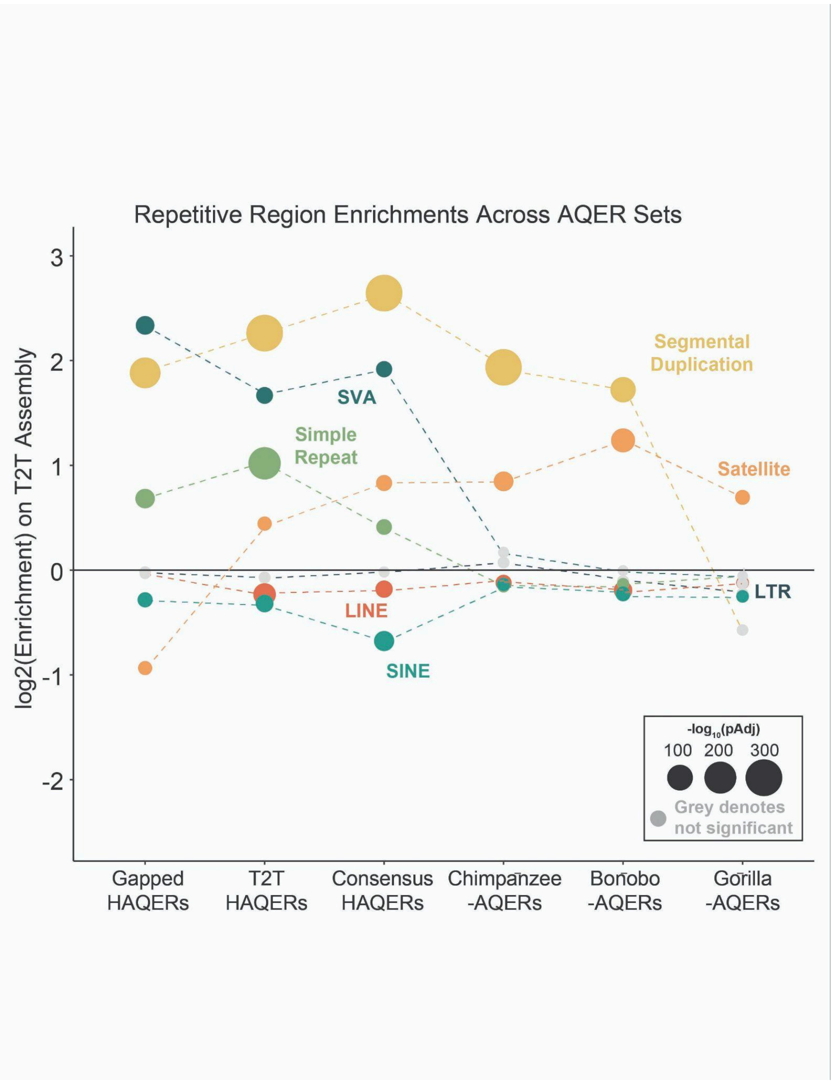

Figure S1: Repetitive region enrichments across HAQER and non-human primate AQER sets, related to Figure 2

Enrichment of HAQER sets and chimpanzee-, bonobo-, and gorilla-AQER sets relative to the genome in major repetitive element classes and families. Ape AQER sets show similar enrichments to HAQERs in segmental duplications and satellite DNA, suggesting similar patterns of rapid evolution across repeat classes across independent primate lineages.

bioRxiv preprint doi: https://doi.org/10.1101/2025.10.20.683446; this version posted October 21, 2025. The copyright holder for this preprint (which was not certified by peer review) is the author/funder, who has granted bioRxiv a license to display the preprint in perpetuity. It is made available under aCC-BY 4.0 International license.

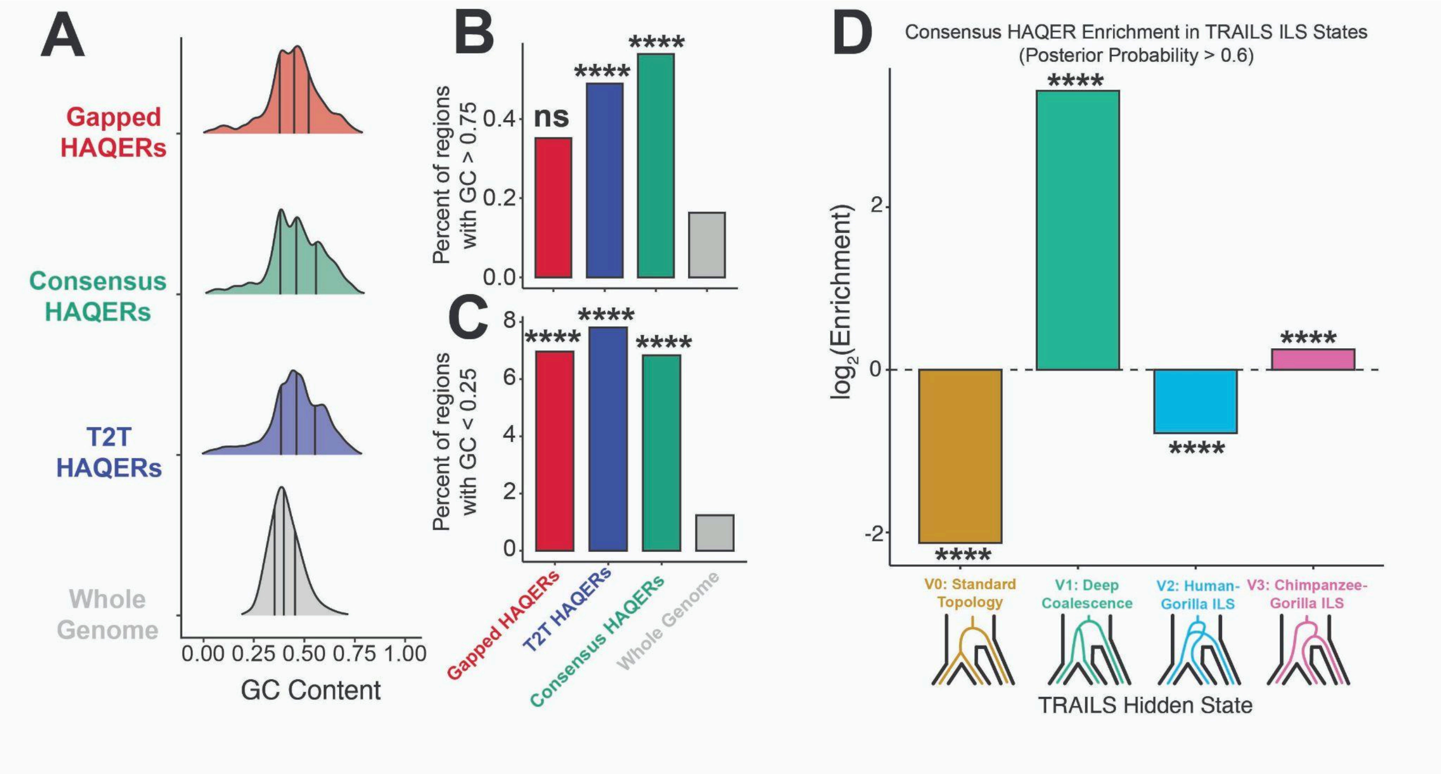

Figure S2: GC content and ILS states of rapidly evolved genomic regions, related to Figure 2

(A) Distribution of GC content across 3 HAQER sets (Gapped HAQERs, T2T HAQERs, Consensus HAQERs) compared with random 500 bp windows sampled from the human genome (Whole Genome).

(B-C) Percentage of (B) GC-rich (GC > 0.75) and (C) GC-poor (GC < 0.25) regions across HAQER sets and the whole genome. Statistical significance was assessed by pairwise chi-squared tests against the whole genome.

(D) Overlap enrichments between Consensus HAQERs and regions of incomplete lineage sorting (ILS) estimated as hidden states of the TRAILS ILS model[43]. Consensus HAQERs are significantly depleted from the V0 state (standard great ape topology) and enriched in the V1 state (standard great ape topology regions with long branches). HAQERs are depleted from the V2 nonstandard topology (human-gorilla ILS) and slightly enriched in the V3 state (chimp-gorilla ILS).

(**** $p < 0.0001$; ns = not significant)

bioRxiv preprint doi: https://doi.org/10.1101/2025.10.20.683446; this version posted October 21, 2025. The copyright holder for this preprint (which was not certified by peer review) is the author/funder, who has granted bioRxiv a license to display the preprint in perpetuity. It is made available under aCC-BY 4.0 International license.

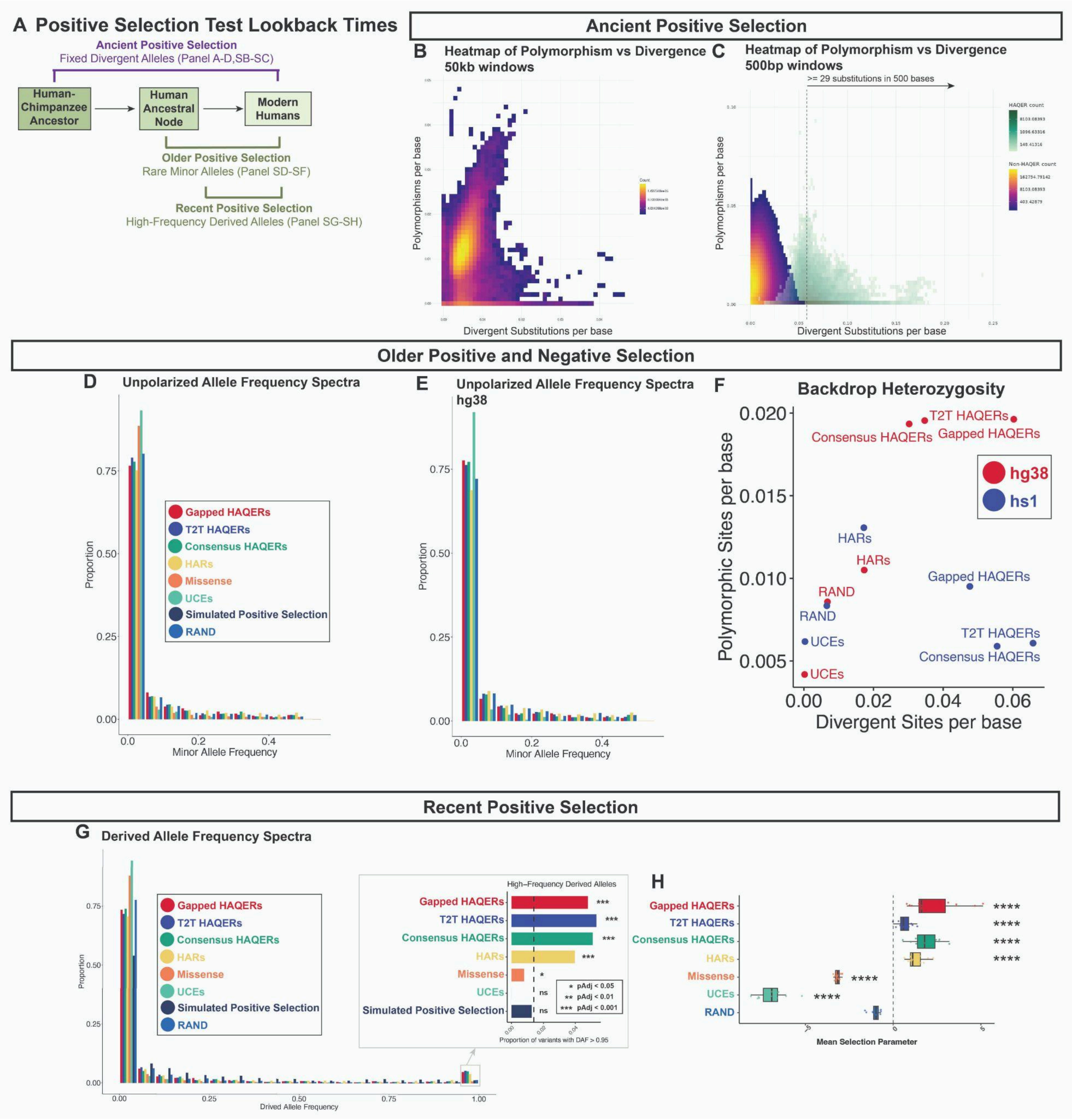

Figure S3: Expanded selection analysis of HAQERs, related to Figure 3

(A) Schematic comparing tests to detect positive selection at different time scales. First, the proportion of fixed divergent alleles detects “ancient” positive selection on the order of millions of years. Ancient beneficial divergences that occurred millions of years ago have enough time to complete selective sweeps, become fixed in the population, and regain polymorphism[60,61]. However, their polymorphism-to-divergence ratio would still be lower than expected due to positive selection. Second, a high proportion of rare minor alleles suggests “older” positive selection, within the past hundreds of thousands of years, enough time for derived beneficial divergences and their nearby linked variants to complete selective sweeps, become fixed in the population, and experience an initial restoration of heterozygosity. Because mutations are rare overall, they restore polymorphism in the positively selected regions slowly. Initially, mutated alleles in the positively selected regions have very low frequencies. At this stage, the low-frequency new mutations are detected as rare minor

bioRxiv preprint doi: https://doi.org/10.1101/2025.10.20.683446; this version posted October 21, 2025. The copyright holder for this preprint (which was not certified by peer review) is the author/funder, who has granted bioRxiv a license to display the preprint in perpetuity. It is made available under aCC-BY 4.0 International license.

alleles. These new alleles are also under negative selection. Given more time, the new alleles will rise in frequency through genetic drift, so that there is no longer an excess of rare minor alleles, and only a low polymorphism-to-divergence ratio remains. Third, the proportion of high-frequency derived alleles detects “recent” positive selection, within the past tens of thousands of years, enough time for beneficial derived alleles and their nearby linked variants to rise to higher frequencies than the ancestral alleles, but not enough time for them to complete selective sweeps and become fixed in the population. The underlying assumption is the infinite sites model, which is incompatible with reversion mutations.

(B-C) Density plot displays the relationship between the number of divergent substitutions and the number of polymorphisms in (B) 50 kb and (C) 500 bp windows along each autosome. Windows that fall within Consensus HAQERs (green) clearly deviate from the genome-wide trend.

(D-E) Unpolarized allele frequency spectra across segregating sites in 501 African individuals (1,002 alleles) within region sets on (D) hs1 or (E) hg38. HAQER sets show elevated proportions of rare minor alleles (minor allele frequency < 0.05) compared to RAND on hg38 but not on hs1.

(F) Scatter plot of divergent sites per base against polymorphic sites per base for each region set, evaluated on either hg38 (red) or hs1 (blue). HAQERs show large differences in polymorphic site estimates across references, consistent with their enrichments in repetitive regions, which may be prone to variant calling misclassification. This suggests that the rare minor allele excess observed in HAQERs in hg38 may be the result of a variant calling artifact.

(G) Derived allele frequency spectra across 501 African individuals (1,002 alleles) for segregating sites within region sets. The 3 HAQER sets have the highest proportions of high frequency derived alleles (derived allele frequency > 0.95). In the inset, the dotted line represents RAND. Statistical significance is assessed by pairwise chi-squared tests against RAND.

(H) Mean selection parameters for each region set, inferred from the segregating sites of the 5 independent populations within the 501 African individuals. Based on our estimations of mean selection parameters, HAQERs and HARs are under positive selection, in contrast with Missense regions and UCEs, which are under negative selection. Statistical significance is assessed by pairwise t-tests against RAND (**** $p < 0.0001$).

All analyses shown were conducted on hs1 unless otherwise stated.

bioRxiv preprint doi: https://doi.org/10.1101/2025.10.20.683446; this version posted October 21, 2025. The copyright holder for this preprint (which was not certified by peer review) is the author/funder, who has granted bioRxiv a license to display the preprint in perpetuity. It is made available under aCC-BY 4.0 International license.

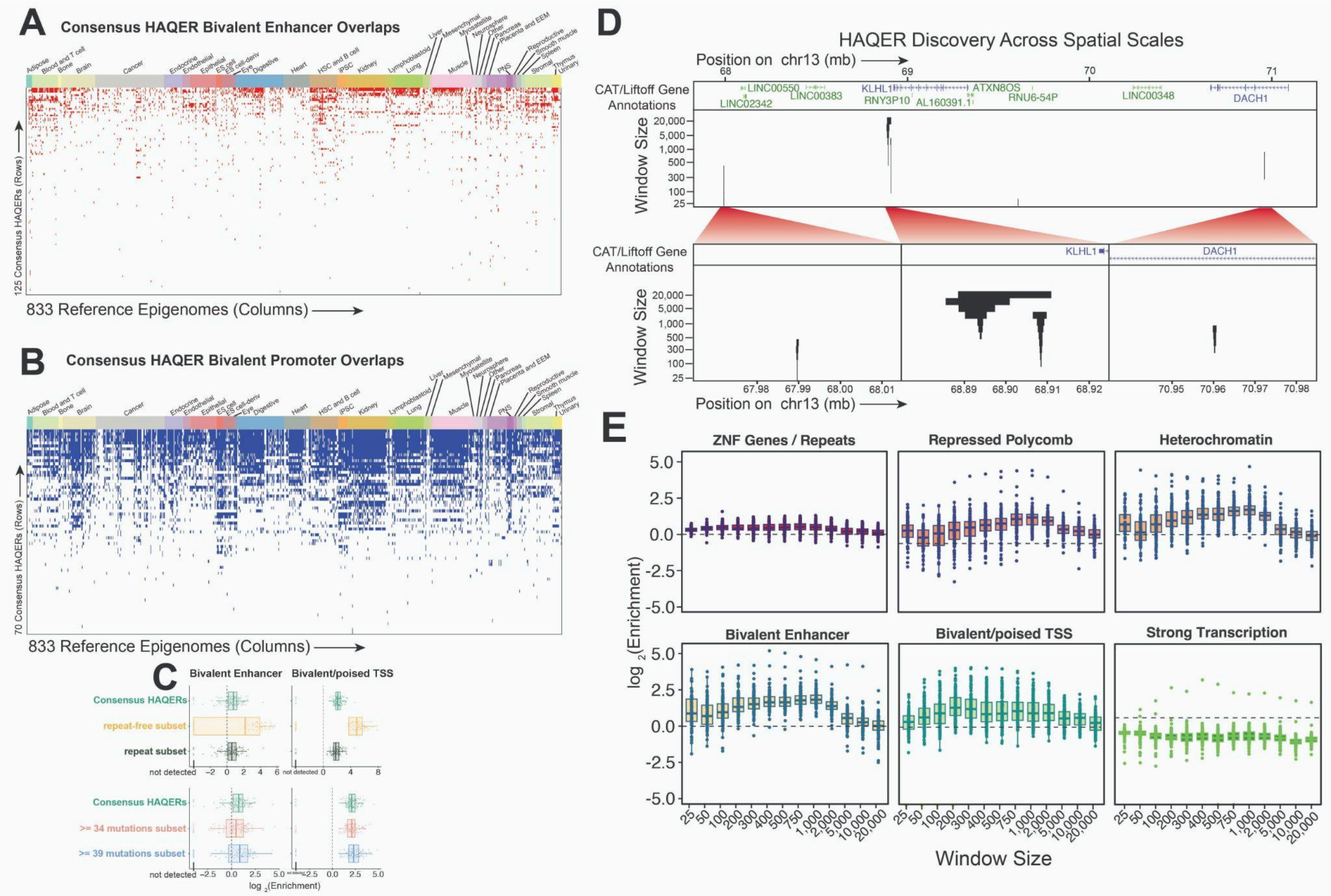

Figure S4: Expanded epigenomic analysis of HAQERs, and multi-scale divergence landscapes of the human genome, related to Figure 4

(A-B) Heatmap of Consensus HAQERs (rows) overlapping (B) bivalent enhancer states and (C) bivalent promoter states across 833 reference epigenomes[17](columns).

(C) Overlap enrichments between bivalent regulatory states (Bivalent Enhancer; Bivalent/poised TSS) across 127 reference epigenomes[62] and Consensus HAQERs, including subsets restricted to HAQERs overlapping repetitive elements (repeat subset) and its complement (repeat-free subset) or subsets defined at higher divergence thresholds (≥34 or ≥39 mutations).

(D) HAQER genomic locations, identified across diverse spatial scales, in a 4 mbp region of chr13. Some larger-scale HAQERs are composite elements, composed of multiple HAQER elements identified at smaller window sizes (middle).

(E) Chromatin state enrichments for T2T HAQERs identified at a range of window sizes across 127 reference epigenomes[62].

bioRxiv preprint doi: https://doi.org/10.1101/2025.10.20.683446; this version posted October 21, 2025. The copyright holder for this preprint (which was not certified by peer review) is the author/funder, who has granted bioRxiv a license to display the preprint in perpetuity. It is made available under aCC-BY 4.0 International license.

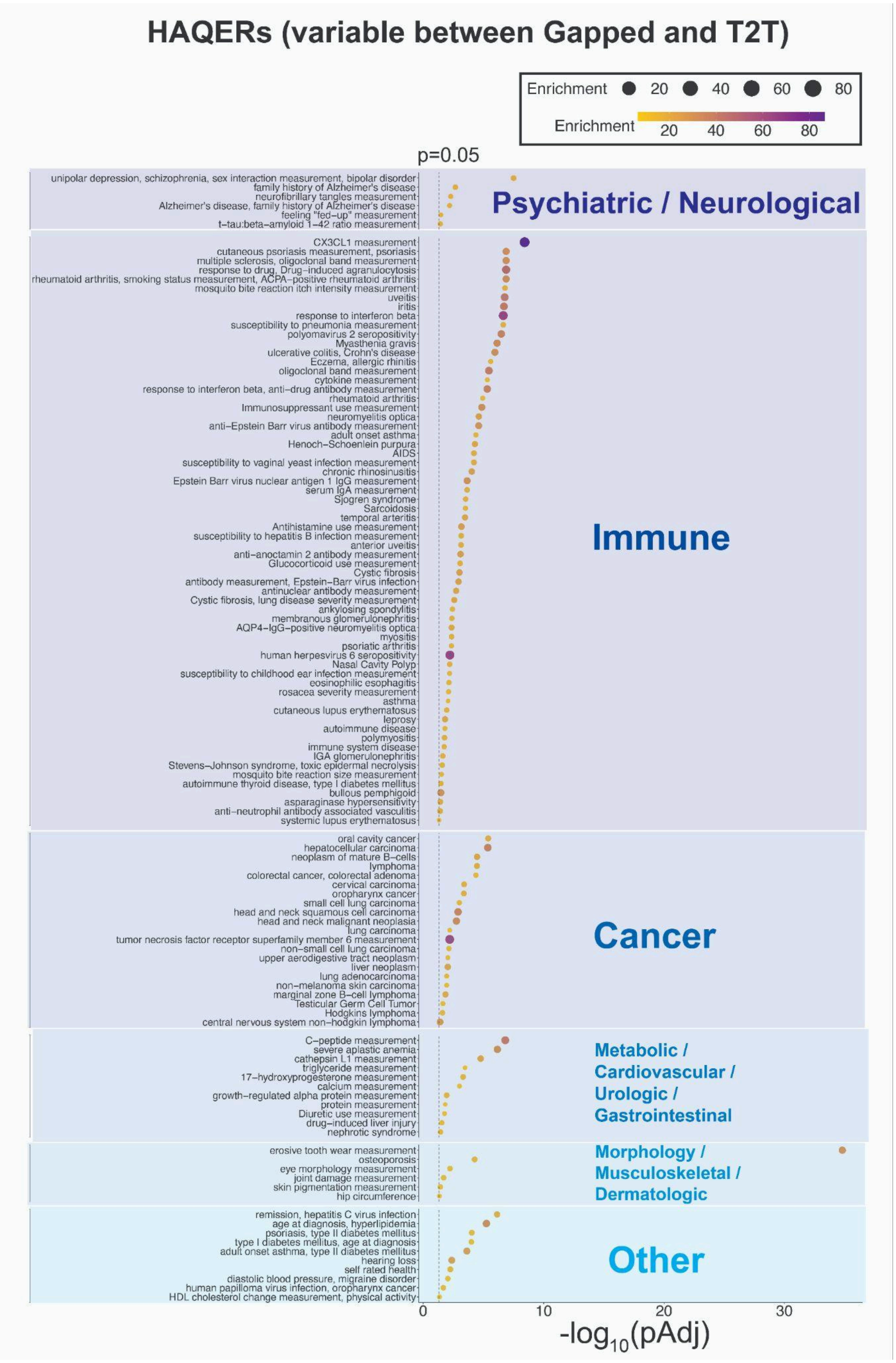

Figure S5: GWAS trait enrichments for variable HAQERs, related to Figure 4

Overlap enrichments between the variable HAQER set - which consists of Gapped HAQERs not overlapping T2T HAQERs and T2T HAQERs not overlapping Gapped HAQERs, and GWAS (genome-wide association study) loci, together with their linked variants (Plink $R^2 > 0.7$). Traits with significant FDR-adjusted enrichments ($p < 0.05$) are shown. Variable HAQERs display widespread enrichments across psychiatric, neurodegenerative, immune, metabolic, and cancer-related traits.

bioRxiv preprint doi: https://doi.org/10.1101/2025.10.20.683446; this version posted October 21, 2025. The copyright holder for this preprint (which was not certified by peer review) is the author/funder, who has granted bioRxiv a license to display the preprint in perpetuity. It is made available under aCC-BY 4.0 International license.

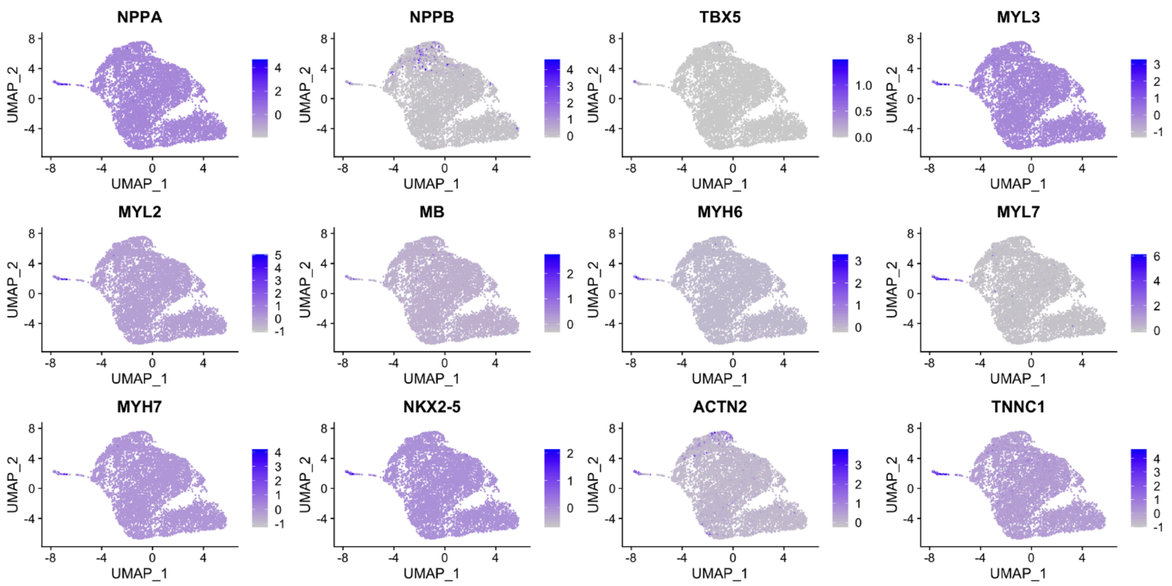

Figure S6: iPSC-derived cardiomyocytes express canonical cardiomyocyte marker genes, related to Figure 5

UMAP projection of iPSC-derived cardiomyocytes highlighting expression of select marker genes, including contractile machinery genes such as myosin light (*MYL2/3/6*) and heavy chains (*MYH6/7*), myoglobin (*MB*), natriuretic peptides (*NPPA* and *NPPB*), and transcriptional and developmental regulators (*NKX2-5* and *TBX5*).
